# Extremely chaotolerant and kosmotolerant *Aspergillus atacamensis* – a metabolically versatile fungus suitable for recalcitrant biosolid treatment

**DOI:** 10.3389/fmicb.2023.1191312

**Published:** 2023-06-28

**Authors:** Tonatiuh Moreno-Perlin, Gisell Valdés-Muñoz, Irina Jiménez-Gómez, Nina Gunde-Cimerman, Luis Andrés Yarzábal Rodríguez, María del Rayo Sánchez-Carbente, Alfaniris Vargas-Fernández, Adrián Gutiérrez-Cepeda, Ramón Alberto Batista-García

**Affiliations:** ^1^Centro de Investigación en Dinámica Celular, Instituto de Investigación en Ciencias Básicas y Aplicadas, Universidad Autónoma del Estado de Morelos, Cuernavaca, Morelos, Mexico; ^2^Department of Biology, Biotechnical Faculty, University of Ljubljana, Ljubljana, Slovenia; ^3^Carrera de Bioquímica y Farmacia, Unidad de Salud y Bienestar, Universidad Católica de Cuenca, Cuenca, Ecuador; ^4^Centro de Investigación en Biotecnología, Universidad Autónoma del Estado de Morelos, Cuernavaca, Morelos, Mexico; ^5^Instituto de Investigación en Salud, Facultad de Ciencias de la Salud, Universidad Autónoma de Santo Domingo, Santo Domingo, Dominican Republic; ^6^Instituto de Química, Facultad de Ciencias, Universidad Autónoma de Santo Domingo, Santo Domingo, Dominican Republic

**Keywords:** *Aspergillus atacamensis*, obligate halophilic aspergilli, xerophiles, salt-adapted fungi, phenotype microarrays, biosolid treatment, chaotolerant and kosmotolerant fungi

## Abstract

Obligate halophily is extremely rare in fungi. Nevertheless, *Aspergillus atacamensis* (strain EXF-6660), isolated from a salt water-exposed cave in the Coastal Range hills of the hyperarid Atacama Desert in Chile, is an obligate halophile, with a broad optimum range from 1.5 to 3.4 M of NaCl. When we tested its ability to grow at varied concentrations of both kosmotropic (NaCl, KCl, and sorbitol) and chaotropic (MgCl_2_, LiCl, CaCl_2_, and glycerol) solutes, stereoscopy and laser scanning microscopy revealed the formation of phialides and conidia. *A. atacamensis* EXF-6660 grew up to saturating levels of NaCl and at 2.0 M concentration of the chaotropic salt MgCl_2_. Our findings confirmed that *A. atacamensis* is an obligate halophile that can grow at substantially higher MgCl_2_ concentrations than 1.26 M, previously considered as the maximum limit supporting prokaryotic life. To assess the fungus’ metabolic versatility, we used the phenotype microarray technology Biolog FF MicroPlates. In the presence of 2.0 M NaCl concentration, strain EXF-6660 metabolism was highly versatile. A vast repertoire of organic molecules (~95% of the substrates present in Biolog FF MicroPlates) was metabolized when supplied as sole carbon sources, including numerous polycyclic aromatic hydrocarbons, benzene derivatives, dyes, and several carbohydrates. Finally, the biotechnological potential of *A. atacamensis* for xenobiotic degradation and biosolid treatment was investigated. Interestingly, it could remove biphenyls, diphenyl ethers, different pharmaceuticals, phenols, and polyaromatic hydrocarbons. Our combined findings show that *A. atacamensis* EXF-6660 is a highly chaotolerant, kosmotolerant, and xerotolerant fungus, potentially useful for xenobiotic and biosolid treatments.

## Introduction

Among the conditions considered “extreme” for life, the presence of high concentrations of salts stands out. Indeed, when microbial cells grow in saline or hypersaline environments, they must face a reduced availability of water (low water activity), which is a major challenge for life. Since cell membranes are permeable to water, the low water activity (a_w_) in saline environments promotes a constant water efflux from the cell to the external medium and, thus, microorganisms present in these types of environments must fight dehydration continuously (Gunde-Cimerman et al., 2018). While many microbial species are not able to cope with such conditions and, therefore, die, some microorganisms evolved different strategies to maintain the osmotic balance of the cell, allowing them not only to survive, but to multiply actively in the presence of high salt concentrations (Gunde-Cimerman et al., 2018).

Even though the term is subject to a certain debate, *obligate halophiles* are a heterogeneous group of microorganisms that require some level of salt, in the form of sodium ions –NaCl (3%, ≅ 0.5 M), for them to grow (Oren, 2008). Although several yeasts –such as *Aureobasidium pullulans, Hortaea werneckii* (Gunde-Cimerman et al., 2018), *Hyphopichia burtonii*, and *Hyphopichia pseudoburtonii* (Lee et al., 2021)– and certain filamentous fungi –such as *Aspergillus ruber* (previously *Eurotium rubrum*) (Kis-Papo et al., 2014), *Aspergillus sclerotialis* (Tafer et al., 2019), and *Aspergillus sydowii* (Jiménez-Gómez et al., 2020, 2022; Pérez-Llano et al., 2020a)– have shown high halotolerance, the obligate halophily is extremely rare in fungi. To date, only the filamentous basidiomycetous *Wallemia ichthyophaga* and *Wallemia muriae* (Zalar et al., 2005; Zajc et al., 2014a,b), *Aspergillus baarnensis* (=*Basipetospora halophila*)*, Aspergillus salinarus* (Greiner et al., 2014), *Aspergillus destruens* (Sklenář et al., 2017), and the phialosimplex-like *Aspergillus salisburgensis* and *Aspergillus atacamensis* (Martinelli et al., 2017) have been described as obligate halophiles, because they need 5–20% NaCl to grow.

Extremely halotolerant microbes, including obligate halophiles, are frequently polyextremophiles, meaning that they are also able to cope with other extreme conditions (Gostinčar et al., 2022), such as extreme values of pH (Mesbah and Wiegel, 2012) and temperatures (Mavromatis et al., 2009), and high doses of UV and ionizing radiation (Babič et al., 2018), among others. Also, the ability of certain fungi to grow at extremely high concentrations of NaCl (> 1.0 M) is accompanied by their capacity to populate environments dominated by other salts such as CaCl_2_, KCl, MgCl_2_, and MgSO_4_, among others (Zajc et al., 2014a; Gostinčar et al., 2022). For instance, the most halophilic fungus known to date, *W. ichthyophaga*, can grow in saturated NaCl, KCl and MgSO_4_ solutions, while the polyextremotolerant black yeast-like fungus *Exophiala dermatitidis* is able to multiply at high concentrations of several salts, such as CaCl_2_, KCl, MgCl_2_, MgSO_4_, NaBr, and NaCl (Gostinčar et al., 2022). Nevertheless, the knowledge concerning fungal growth at extremely high concentrations of various stabilizing (kosmotropic) and destabilizing (chaotropic) salts is still scarce.

The obligate halophile fungus *A. atacamensis* strain EXF-6660 was isolated from a salt water-exposed cave in the Coastal Range hills of the hyperarid Atacama Desert. Interestingly, this strain was recovered from a biofilm present on the cave wall, an extreme xeric environment (Martinelli et al., 2017). *A. atacamensis* EXF-6660 grows optimally at concentrations ranging from 10 to 15% NaCl (≅ 1.7 M – 2.5 M) and can endure up to 25% (≅ 4.3 M) of NaCl (Martinelli et al., 2017). When grown under optimal conditions, *A. atacamensis* exhibits acid phosphatase, amylolytic, esterase, galactosidase, glucosidase, lipolytic, and trypticase activities, but also α-aminoacyl-peptide hydrolase (arylamidase) activity, able to catalyze the hydrolysis of N-terminal amino acids (i.e., cysteine, leucine, and valine) from peptides (Martinelli et al., 2017).

The ability of obligate halophiles to thrive under these conditions has raised the interest of scientists in the last decade, because of their potential use (and that of their biomolecules) in many fields such as agriculture, medicine, and biotechnology. Examples include halostable enzymes produced by halophilic fungi, some of which have the potential to be adopted by the industry for a wide range of applications in high-salt concentrations, a condition that typically inhibits other known enzymes (Śliżewska et al., 2022). However, the metabolic versatility and biotechnological potential of *A. atacamensis* remain unknown.

In the present study, we aimed to explore the morphological and metabolic changes of *A. atacamensis* EXF-6660 when grown in the presence varying concentrations of different solutes (i.e., CaCl_2_, KCl, LiCl, MgCl_2_, NaCl, sorbitol, and glycerol). We first investigated the response of the fungus to different concentrations of chaotropic and kosmotropic salts, and organic solutes. We then explored the utilization of 95 different substrates, by *A. atacamensis* EXF-6660, under four different conditions of salinity (absence, optimal, sub-optimal, and saturating). We also evaluated the ability of *A. atacamensis* EXF-6660 to metabolize different xenobiotic compounds (polycyclic aromatic hydrocarbons (PAHs), benzene derivatives, and dyes) and several carbohydrates, to test its potential for bioremediation and ecological restoration. Finally, we demonstrated that the obligate halophilic *A. atacamensis* EXF-6660 shows high potentialities for biosolid transformation. This is the most comprehensive study to date about the morphology and metabolism of an extremely chaotolerant and kosmotolerant fungus.

## Materials and methods

### Strain source and preservation

The obligate halophilic ascomycete *A. atacamensis* strain EXF-6660, isolated from a wall biofilm from a salt water-exposed cave about 106 km south of Iquique city in the Coastal Range hills of the hyperarid Atacama Desert in Chile (coordinates: 21°12′47″S, 70°05′30″W), was used in this work (Martinelli et al., 2017). *A. atacamensis* EXF-6660 was obtained from the Ex Microbial Culture Collection of the Infrastructural Centre Mycosmo (MRIC UL), at the Department of Biology, Biotechnological Faculty, University of Ljubljana (Slovenia). Fungal cultures were propagated on Malt Extract Agar (MEA composition per liter: malt extract 10 g, dextrose 10 g, mycological peptone 1 g, agar 20 g) or Malt Extract Broth (MEB) supplemented with 1.7 M NaCl. Mycelia and spores were preserved at both 4°C and −80°C in 20% glycerol. Spores and mycelia obtained from 7-day-old cultures of *A. atacamensis* EXF-6660, grown in MEB supplemented with 1.7 M NaCl, were used as pre-inoculum in all experiments.

### Morphology of *Aspergillus atacamensis* EXF-6660 in the presence of different solutes

To evaluate the macro- and micro-morphological characteristics of *A. atacamensis* EXF-6660 in the presence of different solutes, 100 μg of mycelium was plated over MEA supplemented with either CaCl_2_, KCl, LiCl, MgCl_2_, NaCl, glycerol or sorbitol. Different concentrations (3, 5, 5.8, 8.7, 10, 12, 15, 20, 25, and 30% *w/v*) of each solute were tested.

Macro-morphological characteristics, such as shape, elevation, texture, and colony edge, were analyzed from 30-day-old cultures of *A. atacamensis* incubated at 28°C in the presence of different salts and solutes. Fungal cultures were observed using an AmScope stereo microscope with FMA050 fixed adapter and an AmScope MU1000 coupled camera, with 0.7× and 2× objectives (AmScope, Irvine, CA, United States). The microculture technique was used for the micro-morphological characterization of *A. atacamensis*. An AmScope T720 brightfield microscope (AmScope, Irvine, CA, United States) coupled to an AmScope MU1000 series camera with 40× and 100× objectives was used to analyze hyphae, branching, and reproductive structures. Also, the inverted agar block method (Hickey and Read, 2003) was used for micro-morphological observations of *A. atacamensis* EXF-6660, using a live-cell imaging system equipped with an inverted laser scanning microscope (AxioObserver Z.1, Zeiss) with an Apo 100× O/1.4 objective. In all cases, thirty independent hyphae were analyzed. Figures were processed and produced using Adobe Photoshop CS6 Extended (Adobe Systems Inc., San Jose, CA, United States).

### High throughput metabolic profiling using Biolog technology

High throughput metabolic profiling was used to characterize the metabolic versatility of *A. atacamensis* EXF-6660 at different NaCl concentrations (0, 0.85, 2.0, and 5.13 M). Biolog FF (filamentous fungi) MicroPlate^™^ panels (Biolog, Hayward, CA, United States) were used to investigate the fungal respiration in the presence of 95 different organic compounds including acids, amines, amino acids, amino sugars, glucosides, nucleosides, polyalcohols, and sugars. For the metabolic fingerprint analysis, a 7-day-old mycelium of *A. atacamensis* was initially washed with PBS 1X to remove the excess of nutrients derived from fungal cultures in MEB. Later, 2 mL of the mycelium were transferred to a sterile 15 mL Falcon tube, where the mycelium was thoroughly macerated. Afterwards, 10 mL of the inoculation fluid supplied by Biolog company and NaCl at different final concentrations of 0, 0.85, 2.0, and 5.13 M, were added to the tube containing the mycelium. This suspension was filtered using a 40 μm cell strainer. Mycelium aliquots of 0.2 OD_750_ were obtained. Hundred microliters of the mycelium suspension were inoculated into each well of the FF MicroPlate according to the Biolog instructions. FF MicroPlates were incubated at 28°C for 25 days and monitored spectrophotometrically at both 490 and 750 nm to determine the cellular respiration and hyphal density, respectively (Frac et al., 2016; Chou et al., 2022). FF MicroPlates were read using a Synergy H1 hybrid reader and accompanying Gen5 v.1.11 software package (BioTek, Winooski, VT, United States). Wells showing a purple color (≥0.2 DO_490 nm_), as result of the Redox tetrazolium reduction, were considered as positive for the particular metabolic reaction; on the contrary, wells that remained colorless were considered as negative for the specific metabolic reaction monitored (Bochner, 2009). Non-inoculated FF MicroPlates were also implemented, as negative controls.

### Agar plate-based screening

To explore the biotechnological potentialities of the obligate halophilic *A. atacamensis* EXF-6660, the fungal growth on different PAHs, benzene derivatives, dyes, and carbohydrates, supplied as unique carbon sources, was evaluated using an agar plate-based screening in the presence of both 0.85 and 2.0 M of NaCl. PAHs such as 2,3-diaminonaphthalene, anthracene, fluorene, naphthalene, *p*-benzoquinone, phenanthrene, and pyrene, as well as benzene derivatives such as benzene butyl phthalate, dioctyl phthalate, gallic acid, guaiacol, phenol, and piperonyl butoxide were used at final concentration of 50 mg/L. PAHs were previously dissolved in acetone before addition to the Petri dishes. Dyes such as anthraquinone, Coomassie brilliant blue G250, Congo red, Remazol brilliant blue R, and Safranin were also used, at final concentrations of 5 mg/L each. Carbohydrates such as cellulose, chitosan, cornstarch, lignin, maltose, and xylose were also supplied to the cultures as sole carbon sources, at 2% (*w/v*).

Inoculation of plates was performed with 7-day-old mycelium of *A. atacamensis* EXF-6660 previously macerated, homogenized, and washed with PBS 1X. Petri dishes containing mineral medium, supplemented with the different tested compounds, were inoculated with 15 μL of mycelium suspensions filtered using a 40 μm cell strainer and incubated for 10 days at 28°C. The composition of the mineral medium was: 7.8 mg/L CuSO_4_.5H_2_O, 18 mg/L FeSO_4_.7H_2_O, 500 mg/L MgSO_4_.7H_2_O, 10 mg/L ZnSO_4_.7H_2_O, 50 mg/L KCl, 1 g/L K_2_HPO_4_, 2 g/L NH_4_NO_3_, 2 g/L KH_2_PO_4_, 100 mg/L CaCl_2_, 5 mg/L MnSO_4_, 0.1 mg/L H_3_BO_3_, 0.1 mg/L NaMoO_4_.2H_2_O, and 1 mg/L CoCl_2_. Fungal cultures in mineral medium, with no organic compounds added, were also inoculated and incubated under the same conditions, and were used as controls in all above tests. Three replicates of each culture were analyzed in each case. The area of the colonies, expressed in mm^2^, was calculated using ImageJ software. AmScope MU1000 stereoscope (AmScope, Irvine, CA, United States) equipped with a FMA050 adapter and an AmScope MU1000 camera was used for data acquisition in all cases. Photos were taken on the fifth and tenth day of fungus inoculation.

### Biosolid mycotreatment

Twenty grams of dry solid weight biosolids derived from a wastewater treatment plant were utilized in 250 mL Erlenmeyer flasks for solid-phase experiments. Simple pellet mycelium form was prepared according to Haroune et al. (2014). For that, 3 g of blended mycelium suspension of *A. atacamensis* EXF-6660 previously grown in MEB were added to each solid-phase treatment, which were incubated for 15 days at 28°C, periodically homogenized. Humidity was automatically controlled and was maintained at 15% during the experiments. Triplicate treatments were performed. Uninoculated sludges were used as controls -untreated biosolids. All the analyses were determined at final-point.

Pharmaceutical active compounds, such as amlodipine, atenolol, atorvastatin, bezafibrate, carbamazepine, diazepam, diclofenac, fenofibrate, glibenclamide, hydrochlorothiazide, ibuprofen, indomethacine, ketoprophen, mefenamic acid, naproxen, phenazone, sulfamethazine, sulfapyridine, sulfathiazole, were quantitatively determined according to Haroune et al. (2014) using an Acquity Ultra-Performance liquid chromatography system (Waters Corporation, Milford, MA, United States) equipped with a TQ mass spectrometer and an Acquity HSS T3 column (50 mm × 2.1 mm i.d., 1.7 μm particle size). Quantitative determinations of biphenyls, such as 2,4,4′-trichorobiphenyl, 2,2′,5,5′-tetrachlorobiphenyl, 2,2′,4,5,5′-pentachlorobiphenil, 2,2′,4,4′,5,5′-hexachlorobiphenyl, and 2,2′,3,4,4′,5,5′-heptachlorobiphenyl, and PAHs, such as fluorene, naphthalene, phenanthrene, and pyrene, were performed according to methods EPA-8082 and EPA-8270C. The removal of Tri-BDE-28 (2,4,4′-tribromodiphenyl ether), Tetra-BDE-47 (2,2′,4,4′-tetrabromodiphenyl ether), Penta-BDE-99 (2,2′,4,4′,5-pentabromodiphenyl ether), Penta-BDE-100 (2,2′,4,4′,6-pentabromodiphenyl ether), HBB (hexabromobiphenyl), and PBEB (pentabromoethylbenzene) was determined according to methods described in Rodríguez-Rodríguez et al. (2012). Total phenol content was analyzed as previously reported in Batista-García et al. (2017). Chemical oxygen demand (COD), total dissolved solids (TDS), and total suspended solids (TSS) were determined using Standard Methods part 2540 (Rice et al., 2017).

### Model fitting and statistical analyses

To analyze the differences on the morphological characteristics of *A. atacamensis* EXF-6660, grown in the presence of 1.7 M and 5.13 M NaCl and 1.7 M LiCl, we used a one-way analysis of variance (ANOVA) with a Sidak’s *post hoc* analysis (significance level set at 0.05).

For phenotype microarrays, the Biolog FF MicroPlate data was corrected by subtracting the absorbance value, at day zero, to the absorbance measured for each substrate into each well. This data was used to fit a three parameters sigmoid function using non-linear regressions. The calculated parameters, i.e., growth and consumption kinetics, were used to perform a Redundancy Discriminant Analysis (RDA) using the salinity concentration and the carbon source as explanatory independent variables. We used linear regressions to compare the correlation between the substrate consumption and fungal growth.

To compare the growth of the fungi in the agar-based screening we used one-way ANOVA followed by Tukey’s *post-hoc* test (significance level set at 0.05).

All the mathematical and statistical analyses were performed using the statistical software R version 4.2.2 (R Core Team, 2022) with the aid of the RStudio IDE (version 2022.07.1).

## Results

### Microscopic analysis of *Aspergillus atacamensis* EXF-6660 under different culture conditions

**Figure 1:**
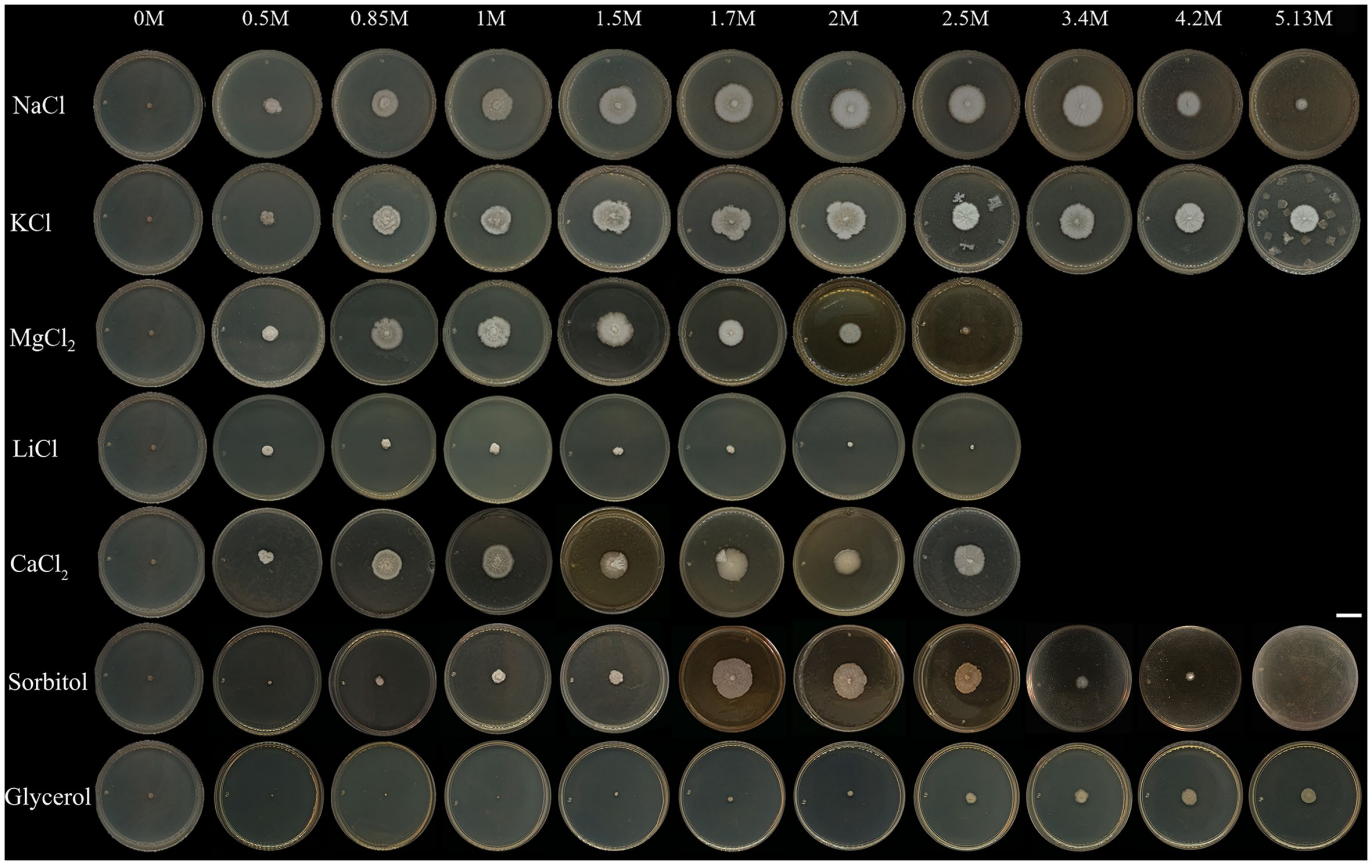
Colonies of *Aspergillus atacamensis* EXF-6660 grown on Malt Extract Agar medium supplemented with the chaotropic salts CaCl_2_, LiCl, and MgCl_2_, the kosmotropic salts NaCl and KCl, sorbitol, or glycerol at different final concentrations: 0, 0.5, 0.85, 1.0, 1.5, 1.7, 2.0, 2.5, 3.4, 4.2, and 5.13 M. Cultures were grown at 28°C for 30 days. Scale bar = 10 mm.

*Aspergillus atacamensis* EXF-6660 grew in the presence of all salts and solutes evaluated at concentrations reducing the a_w_ in the range of 0.9 to 0.85, and even at saturating concentrations of both kosmotropic salts tested, KCl and NaCl. However, macro-morphological analysis of *A. atacamensis* EXF-6660 colonies revealed marked structural changes such as size, color, elevation, type of margin, topography, and texture (Table 1) after 30 days of growth in the presence of different salts and solutes (Figure 1). The highest colony radial growth, evaluated as the colony diameter at the end of the experiment, was recorded in the presence of 1.7 to 2.0 M of the kosmotropic salts KCl and NaCl, as well as in the presence of the same concentration of sorbitol, with colonies reaching 18–20 mm in diameter (Figure 1 and Table 1). Higher or lower concentrations of these solutes yielded smaller colonies, with reduced diameters. In the presence of the chaotropic salts CaCl_2_ and MgCl_2_, the colonies grew at a lesser rate and attained the highest diameters (15–19 mm) at concentrations of 1.5 and 1.7 M, respectively (Figure 1 and Table 1). Noticeably, the presence of glycerol or the weakly chaotropic salt LiCl in the culture medium did not promote *A. atacamensis* EXF-6660 growth to the same degree recorded in the presence of other solutes. Even though it was able to grow in the presence of these two solutes, at concentrations ranging from 0.5 to 1.7 M LiCl, and from 2.5 to 5.13 M glycerol, the colonies were smaller (5–9 mm diameter) than in the presence of other salts or solutes.

**Table 1:** Morphological characteristics of *Aspergillus atacamensis* EXF-6660 in the presence of different concentrations of NaCl, KCl, MgCl_2_, LiCl, CaCl_2_, sorbitol, and glycerol after 30 days of growth at 28°C on Malt Extract Agar.

	NaCl	KCl	MgCl_2_	LiCl	CaCl_2_	Sorbitol	Glycerol
Optimal molarity	1.7 M – 3.4 M	1.5 M – 2.0 M	1.5 M – 1.7 M	1.0 M	0.85 M – 1.7 M	1.7 M – 2.0 M	4.2 M
Diameter	20 mm	18 mm	15 mm	5 mm	16 mm	18 mm	9 mm
Color	White	White-gray	White	White	White-gray	White-gray	Yellow
Elevation	0.5 M Convex	0.5 M Convex	0.5 M Convex	Convex	0.5 M Convex	0.5 M – 1.5 M Convex	0.5 M – 2.0 M Convex
	0.85 M – 2 M Umbonate	0.85 M – 2.0 M Flat	0.85 M – 2.0 M Flat		0.85 M – 2.5 M Flat	1.7 M – 2.5 M Flat	2.5 M – 5.13 M Flat
Margin	0.5 M – 0.85 M Entire	0.5 M – 2.0 M Serrate	0.5 M Entire	Entire	0.5 M – 0.85 M Entire	Entire	Entire
	1.0 M Serrate	2.5 M – 5.13 M Filiform	0.85 M – 1.5 M Serrate		1.0 M Circular		
	1.5 M – 5.13 M Filiform		1.7 M – 2.0 M Filiform		1.5 M – 2.5 M Entire		
Topography		0.5 M – 2.0 M Cerebriform wrinkled	0.5 M – 1.0 M Cerebriform wrinkled	Cerebriform wrinkled	0.5 M – 1.0 M Cerebriform wrinkled	0.85 M – 1.5 M Cerebriform wrinkled	Umbonate
	Radially wrinkled				1.5 M – 1.7 M Umbonate and radially wrinkled		
		2.5 M – 5.13 M Radially wrinkled	1.5 M – 2.0 M Radially wrinkled		2.0 M Umbonate	1.7 M – 2.5 M Umbonate	
					2.5 M Radially wrinkled		
Texture	0.5 M – 1.0 M Granular	0.5 M – 2.0 M Granular	0.5 M – 1.5 M Granular	Granular	0.5 M – 1.0 M, 2.5 M Granular	Granular-Glabrous	Glabrous
	1.5 M – 5.13 M Velvety	2.5 M – 5.13 M Velvety	1.7 M – 2.0 M Velvety		1.5 M – 1.7 M Glabrous-granular		

Differences in the colony elevation were also noticed (Figure 1 and Table 1). For instance, depending on the salt/solute added to the culture medium, the colonies were umbonate (NaCl), umbonate to flat (KCl, CaCl_2_, MgCl_2_, and sorbitol), or convex (LiCl, glycerol). At optimal salt concentrations, the surface of the colonies was granular to glabrous, while under salt stress conditions the surface was mainly glabrous. Even though there was no obvious pigmentation, and the colonies were predominantly white to light grayish, they turned yellow in the presence of glycerol. Further, the mycelium was mainly white, in the center of the colonies, but appeared hyaline in their margins. No exudates and non-pigmented reverse-sides were observed. At suboptimal concentrations, the mycelium appearance was cerebriform, wrinkled and radially wrinkled; on the contrary, at optimal conditions, it turned less wrinkled and even smooth. White conidia were observed in the presence of all solutes; however, kosmotropic salts induced a more abundant conidiation than chaotropic salts. In the absence of any of the salts/solutes tested (0 M), the mycelium turned black, and the fungus died (Figure 1).

**Figure 2:**
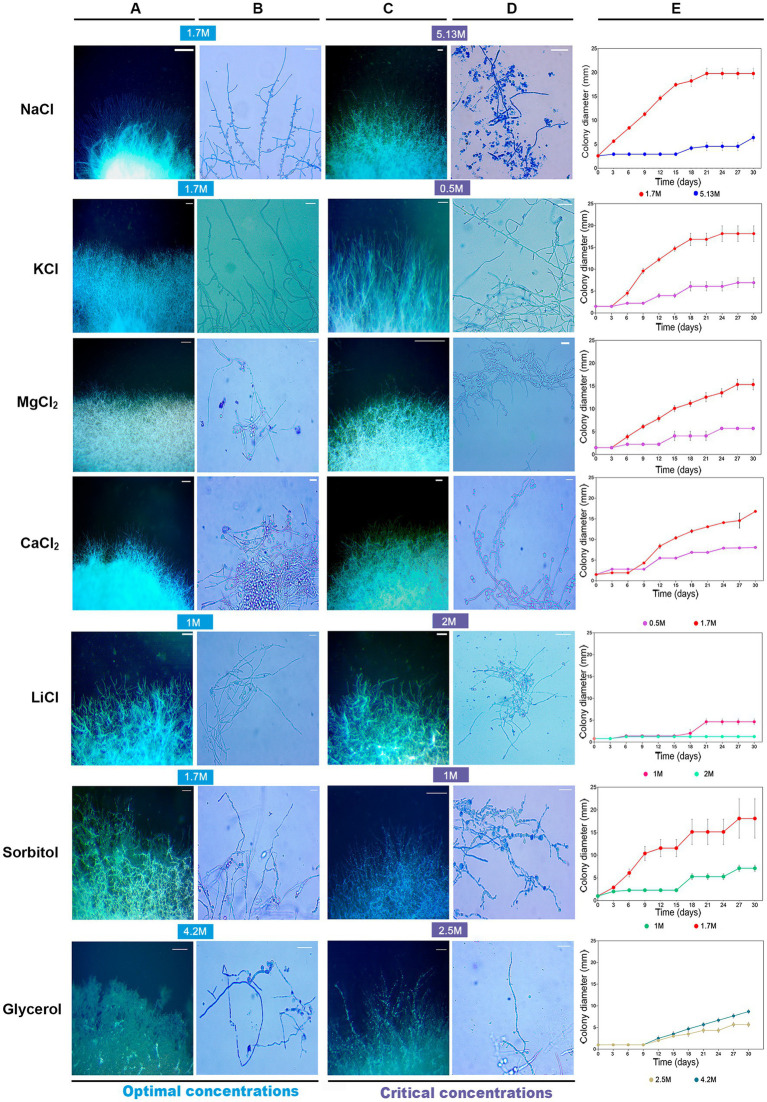
Macroscopic and microscopic features of *Aspergillus atacamensis* EXF-6660 mycelium grown on Malt Extract Agar medium at two different conditions (optimal/critical) for each salt/solute tested: **(A,B)** growth in the presence of optimal salt/solute concentrations and **(C,D)** growth in the presence of critical salt/solute concentrations. NaCl was used at the optimal (1.7 M) and saturated (5.13 M) concentrations since *A. atacamensis* does not grow in the absence of salt. **(A,C)** Macroscopic features. Scale bar = 10 mm. **(B,D)** Microscopic characteristics of hyphae and conidia. Scale bar = 20 μm. **(E)** Colony growth kinetics for 30 days. Optimal concentrations: 1.7 M KCl, MgCl_2_, CaCl_2_ and sorbitol; 1.0 M LiCl; 4.2 M glycerol. Critical concentrations: 0.5 M KCl, MgCl_2_, and CaCl_2_; 2.0 M LiCl; 1.0 M sorbitol; 2.5 M glycerol.

The largest diameter of *A. atacamensis* EXF-6660 colonies was reached in the range 1.5–1.7 M in the case of CaCl_2_, KCl, MgCl_2_, and sorbitol, as well as in the presence of NaCl. The latter observation was expected, since the optimum NaCl concentrations range from 1.5 to 3.4 M as published in a previous study (Martinelli et al., 2017). In the case of LiCl and glycerol, 1.0 and 4.2 M were necessary, respectively, to attain maximal radial growth. We will thus refer to all of the abovementioned concentrations as “optimal growth conditions.” In order to deepen into this subject, we compared micro-morphological traits of *A. atacamensis* EXF-6660 at two different conditions for each salt/solute tested: (i) growth in the presence of optimal salt/solute concentrations (Figures 2A,B) and (ii) growth in the presence of that salt/solute concentration that allowed the minimal growth (critical concentrations). The only exception was NaCl, since we performed this series of experiments using optimal and saturated (5.13 M) NaCl concentrations (Figures 2C,D). Relevant changes were noticed at the macro- and microscopic level (Figures 2A–D). Growth kinetics, evaluated by measuring colony diameter every 3 days for 30 days, confirmed the difference of radial growth under both conditions, optimal and critical concentrations for each salt/solute tested (Figure 2E).

Concentrations that allowed the minimal fungal growth (critical concentrations) induced depolarized hyphal growth and swellings in the presence of certain salts/solutes. This was seen as apical, subapical, or possible vacuolation zones of the hypha indistinctly, except for NaCl and KCl, which did not induce swellings formation (Figure 2B). These swellings fragments were outstanding in MgCl_2_ (0.5 M) and sorbitol (1.0 M). At optimal growth conditions, swellings were also present, but to a lesser degree, in the presence of MgCl_2_, CaCl_2_ and glycerol (Figure 2D).

**Figure 3:**
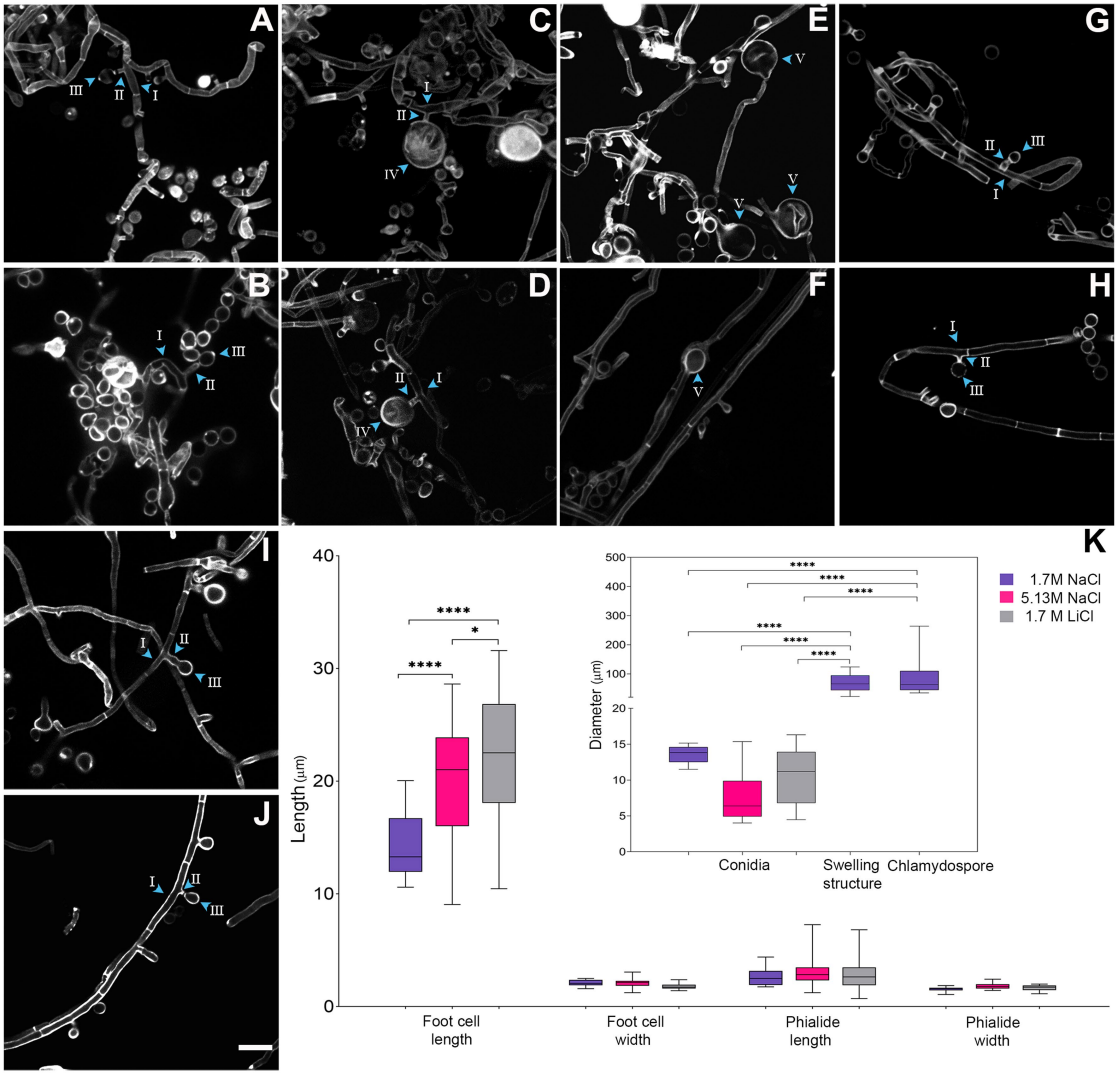
Micro-morphological comparison of *Aspergillus atacamensis* EXF-6660 grown on Malt Extract Agar medium in the presence of 1.7 M NaCl **(A–F)**, 5.13 M NaCl **(G,H)**, and 1.7 M LiCl **(I,J)** staining cell wall with Calcofluor. Scale bars = 100 μm. **(K)** Boxplots representation showing the foot-cell length and width, phialide length and width, conidia diameter and swelling and chlamydospore structure diameter of *A. atacamensis* in the presence of 1.7 M NaCl (violet), 5.13 M NaCl (pink), and 1.7 M LiCl (gray). Arrow heads with roman numbers point toward different structures: I-foot-cell, II-phialide, III-conidia, IV-chlamydospore, V-swelling structure. Sidak’s one-way ANOVA *post hoc* analysis was performed to determine statistical differences (*p* < 0.05). Asterisks indicate statistically significant differences.

To continue with this study, we performed a deeper comparative characterization of the microscopic features of *A. atacamensis* EXF-6660, when grown in the presence of the kosmotropic salt NaCl, considered as the one that promoted optimal mycelial growth, and in the presence of the weakly chaotropic salt LiCl, which showed to be the most toxic tested salt. In the presence of Li-ions, the fungal colonies were distinctly smaller. Significant differences (*p* < 0.05) between the morphological characteristics of *A. atacamensis* grown under three different conditions (1.7 and 5.13 M NaCl, 1.7 M LiCl) were observed (Figure 3).

Average foot-cell width showed no significant differences between conditions while foot-cell length (i.e., distance between two septa) was significantly longer in the presence of LiCl (21.89 μm), as compared to NaCl at both concentrations tested: 5.13 M (19.75 μm) and 1.7 M (14.29 μm) (Figures 3A–K). Phialides were solitary with a terminal locus, lateral, cylindrical, and sometimes tapered or flared toward the hyphal tip (Figures 3A,B,G,J). We also observed solitary conidia or short conidial chains in the presence of NaCl, at both optimal and saturated concentrations (Figures 3B,H). On the contrary, conidial chains were not observed in the presence of the weakly chaotropic salt LiCl (Figures 3I,J). Phialide length, phialide width, and conidia diameter did not differ significantly between conditions (Figure 3K). The most remarkable difference observed, when comparing the micro-morphological features of *A. atacamensis* EXF-6660 under the three different conditions of growth, was the presence of chlamydospores (Figures 3C,D) and swelling cells in the presence of 1.7 M NaCl (Figures 3E,F). Chlamydospores were solitary, thick walled and globose (average diameter 90 μm), usually lateral, arising on short stalks while swellings were usually terminal, thick walled and subglobose to pyriform (average diameter 68 μm). Both chlamydospores and swellings were significantly larger than conidia (Figure 3K).

### High throughput metabolic profiling of *Aspergillus atacamensis* EXF-6660

**Figure 4:**
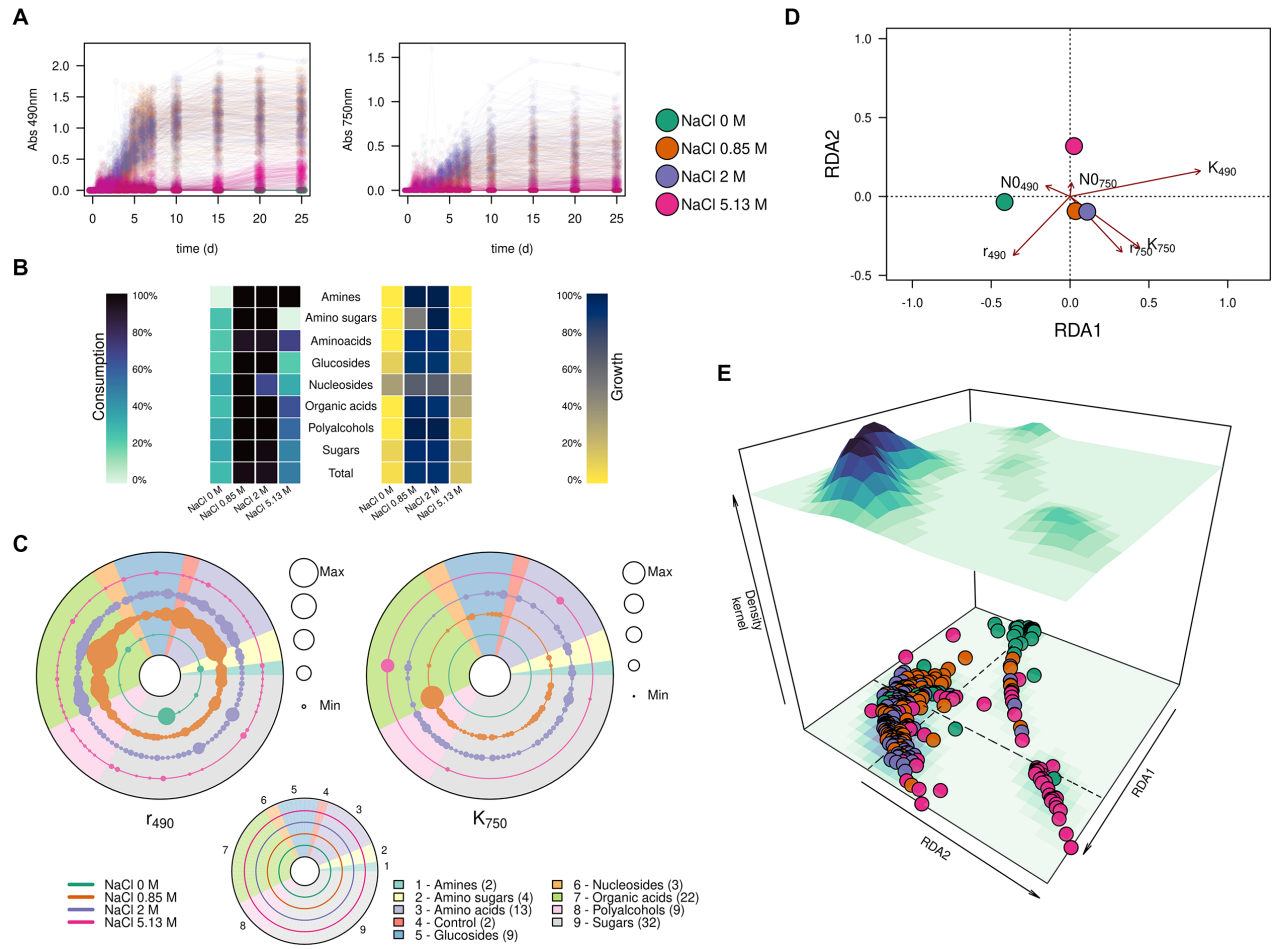
Substrate consumption and growth of *Aspergillus atacamensis* EXF-6660 in the presence of 95 different compounds included in the Biolog FF MicroPlate panels. **(A)** Kinetics of substrate consumption (monitored at 490 nm) and biomass production (monitored at 750 nm) in the absence of any salt (green curves), at optimal (12%, ~ 2.0 M) (purple curves), sub-optimal (5%, ~ 0.85 M) (orange curves) and saturated (30%, ~ 5.13 M) (pink curves) NaCl concentrations. **(B)** Percentages of each group of substrates used for consumption and growth by *A. atacamensis* at different NaCl concentrations. **(C)** Maximum substrate consumption rate (r_490_) and maximum growth (K_750_) of *A. atacamensis* in the presence of different groups of substrates present in the Biolog FF MicroPlates. Solid circles represent the substrates present in the Biolog panel. The size of the circles is proportional to the relative value of the parameter r_490_ or K_750_. Concentric circles represent the different salinity conditions: 0, 0.85, 2.0, and 5.13 M NaCl. The number indicated in parenthesis represents the number of carbon sources for each group of substrates. **(D)** Ordination and **(E)** 3D-density kernel of the ordination of the kinetic parameters related to the curves of substrate consumption and growth of *A. atacamensis* in the presence of the 95 tested substrates.

To provide deeper insights into the physiology of *A. atacamensis* EXF-6660, we analyzed the kinetics of growth and metabolic activity in the presence of 95 different compounds, supplied as unique carbon sources, from three independent liquid cultures obtained in the absence of NaCl and at three different NaCl concentrations: optimal (12%, ~ 2.0 M), sub-optimal (5%, ~ 0.85 M) and saturating (30%, ~ 5.13 M) (Figure 4). The high throughput metabolic profiling of *A. atacamensis* was performed in the presence of the kosmotropic salt NaCl because this fungus was previously described as an obligate halophile (Martinelli et al., 2017).

The first relevant observation is that *A. atacamensis* EXF-6660 can metabolize a heterogeneous structural group of substrates, including organic acids, mono- and polysaccharides, glucosides, polyalcohols, and nitrogen-containing compounds such as amino acids, amino sugars, amines, and nucleosides. This ability to grow, at the expense of these substrates, was hindered by all salinity conditions, even at saturated NaCl concentration. The kinetics of consumption of the 95 different tested compounds, while monitored at saturating salinity conditions (pink curves in Figure 4A), presented a very long lag phase of almost 15 days, as compared to the curves observed at sub-optimal (0.85 M) and optimal (2.0 M) salinity conditions, under which the lag phase lasted less than 3 days (orange and purple curves, respectively, in Figure 4A). As expected, *A. atacamensis* did not grow in the absence of NaCl and its metabolic activity was marginal, with only 28.1% of the substrates partially metabolized under this condition (Figures 4A,B). The consumption of substrates in the presence of optimal (2.0 M NaCl) and sub-optimal (0.85 M NaCl) conditions was accompanied by cellular proliferation. At these salinity conditions *A. atacamensis* EXF-6660 metabolized 94.8 and 96.9%, respectively, of the 95 substrates present in the Biolog FF MicroPlates (Figures 4A,B). At saturated NaCl concentration (5.13 M), even though *A. atacamensis* belatedly consumed almost half (49.5%) of the carbon sources present in the Biolog panels, the fungus showed an erratic growth. In general, the growth of *A. atacamensis* was related to the consumption of ~90% of the Biolog substrates at both optimal and sub-optimal NaCl concentrations; however, the fungal growth was poorly related to the substrate consumption in the absence of salt and at saturated NaCl concentration where only 5.2 and 16.7% of those consumed carbon sources, respectively, were used for growth (Figures 4A,B). Interestingly, although the maximum metabolic activity was observed at 0.85 M NaCl, this was not reflected in the ability of *A. atacamensis* EXF-6660 to grow maximally under the same condition, since growth was slightly lower as compared to 2.0 M NaCl.

To analyze the kinetics of growth of the fungus, data obtained from the Biolog FF MicroPlates was fitted to a logistic model, in which three parameters were considered: the lag phase for substrate consumption or growth (N0), the maximum rate of substrate consumption or growth (r), and the value at which the maximum consumption or maximum growth was reached (k). Maximum substrate consumption rate (r_490_) for each substrate varied drastically in the presence of different NaCl concentrations (see concentric circles in Figure 4C). It proved to be higher at sub-optimal NaCl concentration (5%, ~ 0.85 M), than under any of the other conditions, particularly for organic acids and amino acids (Figure 4C). However, the values at which the maximum growth was reached (K_750_) were higher at the optimal salt concentration (12%, ~ 2.0 M NaCl), indicating that even though more substrates were utilized at a higher rate at the sub-optimal condition, fungal growth was favored at the optimal NaCl concentration, as compared to the other conditions (Figures 4B,C).

We also used the calculated kinetic parameters to perform a redundancy analysis using the salinity and the carbon sources as independent explanatory variables (Figures 4D,E). The ordination analysis indicated that the substrate consumption and growth of *A. atacamensis* at sub-optimal (5%, ~ 0.85 M) and optimal (12%, ~ 2.0 M) NaCl concentrations were very similar (Figures 4C,D). In contrast, these parameters were markedly different in the absence of salt and at saturated NaCl concentration (30%, ~ 5.13 M). Interestingly the results obtained at these two extreme salinity conditions did not cluster together, suggesting that the metabolic behavior of *A. atacamensis* EXF-6660 is different at both ends of the salinity range.

**Figure 5:**
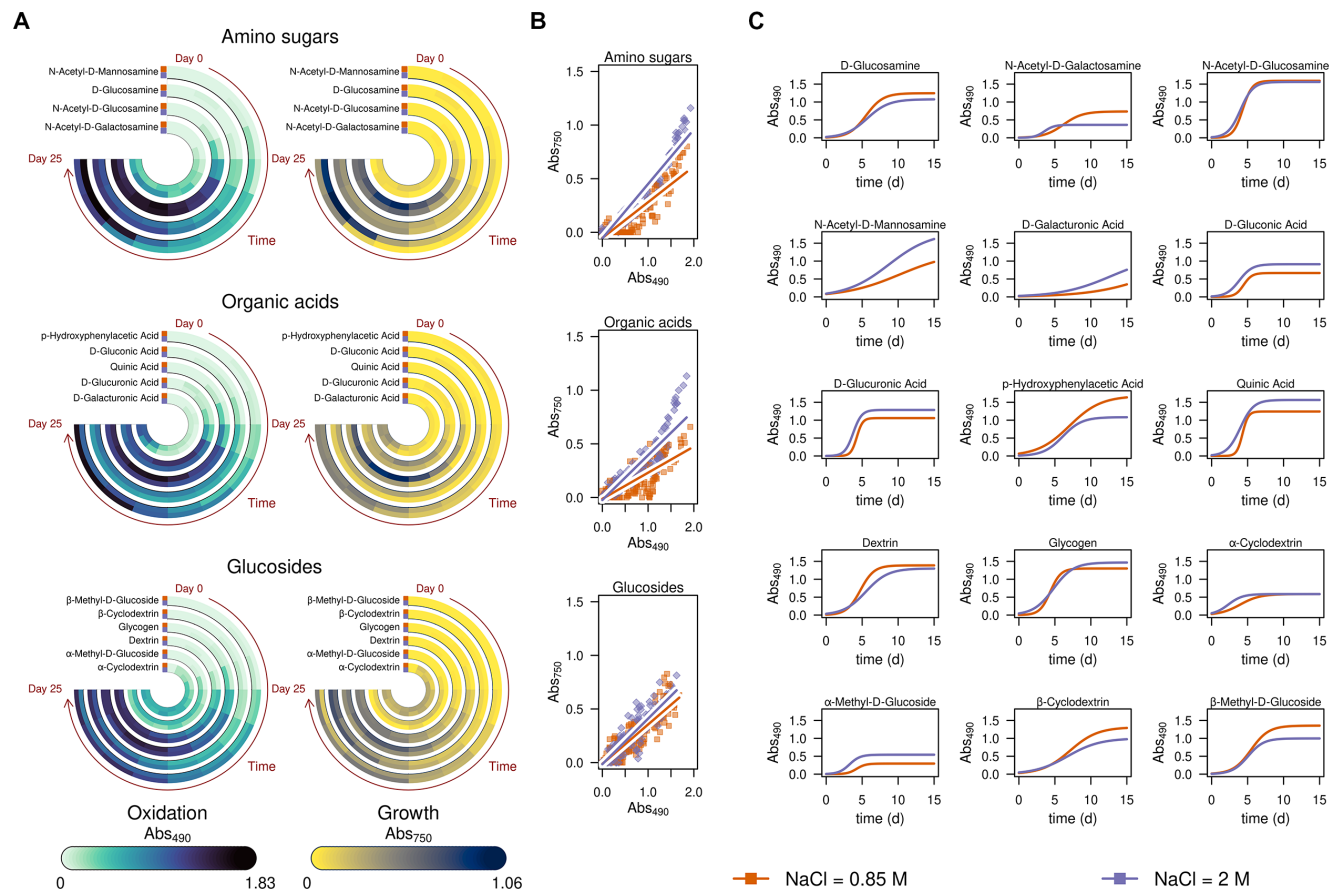
Kinetics of substrate consumption and growth of *Aspergillus atacamensis* EXF-6660 in selected substrates present in the Biolog FF MicroPlates. Data were obtained at optimal (12%, ~ 2.0 M) and sub-optimal (5%, ~ 0.85 M) salinity conditions. **(A)** Substrate consumption monitored at 490 nm and biomass production monitored at 750 nm of *A. atacamensis* grown in the presence of amino sugars, organic acids, and glucosides. **(B)** Correlation of the substrate consumption (abs 490 nm) and growth (abs 750 nm) of *A. atacamensis* at salt concentrations of 0.85 and 2.0 M NaCl in the presence of amino sugars, organic acids, and glucosides. **(C)** Fitted logistic curves of substrate consumption (abs 490 nm) of *A. atacamensis* in the amino sugars, organic acids, and glucosides. Abs, absorbance.

Finally, we further explored the ability of *A. atacamensis* EXF-6660 to metabolize specific carbon sources such as amino sugars, organic acids, and glucosides, at both the optimal and suboptimal salinity conditions. The average substrate utilization and the average observed growth over time in the presence of these carbon sources at both salinities (0.85 and 2.0 M) are shown in Figure 5A. When *A. atacamensis* EXF-6660 was cultured at the optimal NaCl concentration, the fungal growth was not always concomitant with the consumption -degradation- of the substrates. This was the case, for instance, of organic acids such as D-glucuronic and D-gluconic acids, which were consumed quickly by *A. atacamensis* cells, without showing an evident increase in the optical density of the cultures (Figure 5A). On the contrary, consumption of N-acetyl-D-glucosamine and quinic acid was concomitant to an evident growth of the fungal mycelium. Interestingly, a strong difference in the utilization of the glucosides cyclodextrin and methyl-D-glucoside was related to the presence of an α or β glycosidic bond: the utilization of the former anomer was three-fold lower than the consumption of the latter. Moreover, the utilization of these substrates was almost 1.5-times higher at the sub-optimal concentration, as compared to the optimal concentration of NaCl (Figure 5A).

When *A. atacamensis* EXF-6660 was grown at optimal NaCl concentration, the correlation coefficients between the utilization of organic acids or amino sugars and the fungal growth were higher than the ones obtained when cultivated at sub-optimal salinity conditions (Figure 5B), highlighting the requirement of high salt concentrations for optimal growth of this fungus. The same was true for glucosides, except that the difference between the two coefficients was slightly lower (0.87 versus 0.85), an indication that glucosides were substrates that poorly sustained the growth of *A. atacamensis*. When we compared the curves resulting from the fitting of the data obtained from Biolog FF MicroPlate panels to a sigmoid function (Figure 5C), some interesting observations emerged. For instance, in most cases, the curves corresponding to the sub-optimal condition were very similar to the ones obtained from the optimal condition; in some cases, the curves even overlapped (see, for instance, N-acetyl-D-glucosamine). However, the curves remained clearly different and most of them can be distinguished one from another, showing that the kinetic parameters are similar, but not identical, at these salinity conditions. In the case of amino sugars and glucosides, the main differences relate to the asymptotes: for some substrates, the asymptote is reached earlier, at the optimal NaCl concentration (2.0 M), indicating less substrate oxidation (e.g., N-acetyl-D-galactosamine) (Figure 5C). The only exception to this trend seemed to be N-acetyl-D-mannosamine, which appeared to be oxidized better at the optimal NaCl concentration. On the contrary, the consumption of organic acids seemed to occur more rapidly at the optimal salinity condition, as compared to the sub-optimal salinity condition. There was only one exception to this observation: the utilization of *p*-hydroxyphenylacetic acid.

### *Aspergillus atacamensis* EXF-6660 shows a great potential for the biotransformation of xenobiotics and for biosolid treatment

**Figure 6:**
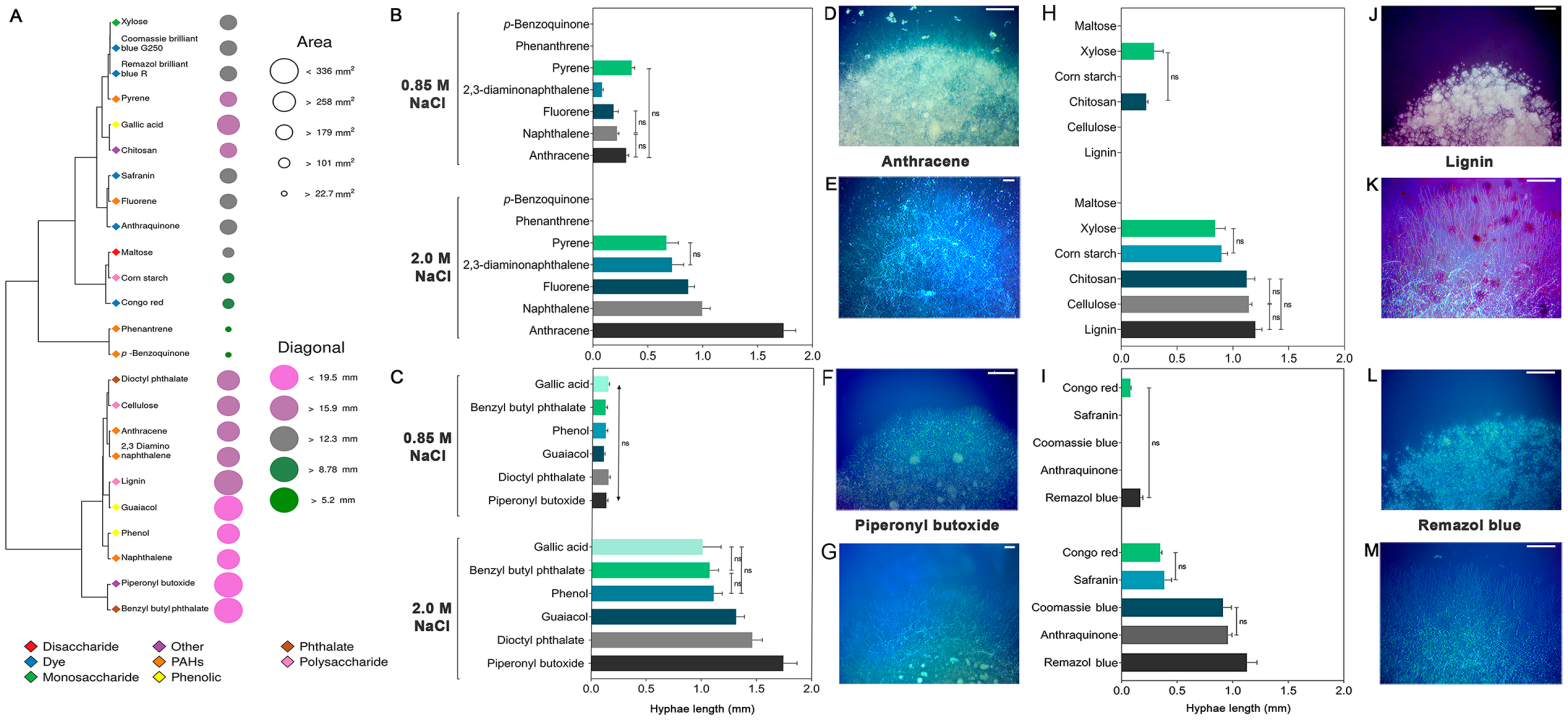
Growth of *Aspergillus atacamensi*s EXF-6660 on mineral medium supplemented with 0.85 and 2.0 M NaCl and a variety of xenobiotics and carbohydrates at 28°C. **(A)** Heat map representing the diameter and area of *A. atacamensis* colonies after 21 days of growth. **(B–M)** Bar graphs and representative pictures showing the length of exploratory hyphae of *A. atacamensis* after 10 days of culture. **(B)** *p*-benzoquinone and polycyclic aromatic hydrocarbons. **(C)** Benzene derivatives. **(H)** Carbohydrates. **(I)** Dyes. **(D,F,J,L)** Colony appearance at 0.85 M NaCl. **(E,G,K,M)** Colony appearance at 2.0 M NaCl. Bars: 500 μm. Representative pictures of fungal growth in the presence of anthracene **(D,E)**, piperonyl butoxide **(F,G)**, lignin **(I,J)**, and Remazol brilliant blue R **(K,L)** as unique carbon sources are shown. One-way ANOVA followed by Tukey’s *post-hoc* test was performed to determine statistical differences.

To investigate the ability of *A. atacamensis* EXF-6660 to metabolize xenobiotic compounds, such as PAHs, benzene derivatives, and dyes, and also carbohydrates, supplied to a mineral medium as the carbon source available, we performed an agar plate-based screening at both 0.85 and 2.0 M NaCl. *A. atacamensis* was able to metabolize almost all carbon sources, except phenanthrene, *p*-benzoquinone and maltose. The fungus showed the highest values of colony area, colony diameter and length of exploratory hyphae in the presence of 2.0 M NaCl (Figure 6).

After 21 days of growth, the resulting colonies at 2.0 M NaCl could be grouped into five categories on the basis of their surface area (Figure 6A): large (area < 336 mm^2^, 15.9 < diagonal<19.5 mm), colonies grown in the presence of benzyl butyl phthalate, guaiacol, lignin, and piperonyl butoxide; medium (area > 258 mm^2^, diagonal >159 mm), colonies obtained in the presence of 2,3-diaminonaphthalene, anthracene, cellulose, dioctyl phthalate, gallic acid, naphthalene, and phenol; small (area > 179 mm^2^, 12.3 < diagonal<15.9 mm), colonies grown in the presence of anthraquinone, chitosan, Coomassie brilliant blue G250, fluorene, pyrene, Remazol brilliant blue R, Safranin, and xylose; minute (area > 101 mm^2^, 8.78 < diagonal<12.3 mm), colonies obtained in the presence of Congo red, cornstarch, and maltose; and finally, punctiform colonies (area > 22.7 mm^2^, diagonal >5.2 mm), colonies grown in the presence of *p*-benzoquinone and phenanthrene.

Among the seven PAHs tested, anthracene promoted the highest growth of fungal exploratory hyphae (average length = 1.92 mm) at 2.0 M NaCl. The average lengths of hyphae in the presence of any of the remaining polycyclic aromatic hydrocarbons were significantly shorter (*p* < 0.05): 1.00 mm (naphthalene), 0.86 mm (fluorene), 0.72 mm (2,3-diaminonaphthalene) and 0.67 mm (pyrene) (Figures 6B,D,E). At 0.85 M NaCl, the hyphae length of *A. atacamensis* was drastically reduced in the presence of the xenobiotics and carbohydrates tested (Figures 6B–M). At this NaCl concentration, exploratory hyphae developed in the presence of several compounds, but their length was drastically reduced as compared to the optimal NaCl concentration. In the presence of 0.85 M NaCl, pyrene allowed the highest growth of exploratory hyphae (average length = 0.35 mm). No fungal growth was observed in the presence of *p*-benzoquinone and phenanthrene (Figure 6B).

In the case of the six benzene derivatives tested, *A. atacamensis* EXF-6660 was able to grow in the presence of all of them at both NaCl concentrations 0.85 and 2.0 M (Figures 6C,F,G). Again, the length of hyphae was significantly greater (*p* < 0.05) at 2.0 M NaCl (average length varied between 1.00 and 1.74 mm, depending on the xenobiotic supplied in the medium), than at 0.85 M NaCl (0.10 to 0.14 mm). Carbohydrate degradation followed the same pattern, with significantly longer hyphae recorded at 2.0 M NaCl than at 0.85 M NaCl (*p* < 0.05). Noticeably, at the sub-optimal salinity condition (0.85 M NaCl) *A. atacamensis* EXF-6660 only grew in the presence of chitosan and xylose, with average hyphal lengths of 0.22 and 0.29 mm, respectively (Figures 6H,J,K). Conversely, and with the only exception of maltose, when tested at 2.0 M NaCl, exploratory hyphae of *A. atacamensis* EXF-6660 developed in the presence of the remaining carbohydrates, reaching their highest values in the presence of lignin (average length = 1.20 mm). Some statistically significant differences were observed between the previously mentioned value and the average lengths of hyphae grown on cellulose (1.14 mm) and chitosan (1.12 mm). The average length of hyphae was significantly lower in the presence of corn starch (0.90 mm) and xylose (0.84 mm), with respect to the other carbohydrates.

Even though *A. atacamensis* EXF-6660 was able to degrade all five dyes at 2.0 M, this activity was severely reduced at 0.85 M NaCl (Figures 6I,L,M). In fact, only Congo red (hyphal average length = 0.07 mm) and Remazol brilliant blue R (hyphal average length = 0.17 mm) were degraded at this salinity. On the contrary, at the optimal NaCl concentration, the fungus grew satisfactorily in the presence of all dyes, exhibiting the longest hyphae in the presence of Remazol brilliant blue R (average length = 1.13 mm), followed by anthraquinone (average length = 0.96 mm), Coomassie brilliant blue G250 (average length = 0.91 mm), Safranin (average length = 0.38 mm), and Congo red (average length = 0.35 mm).

**Figure 7:**
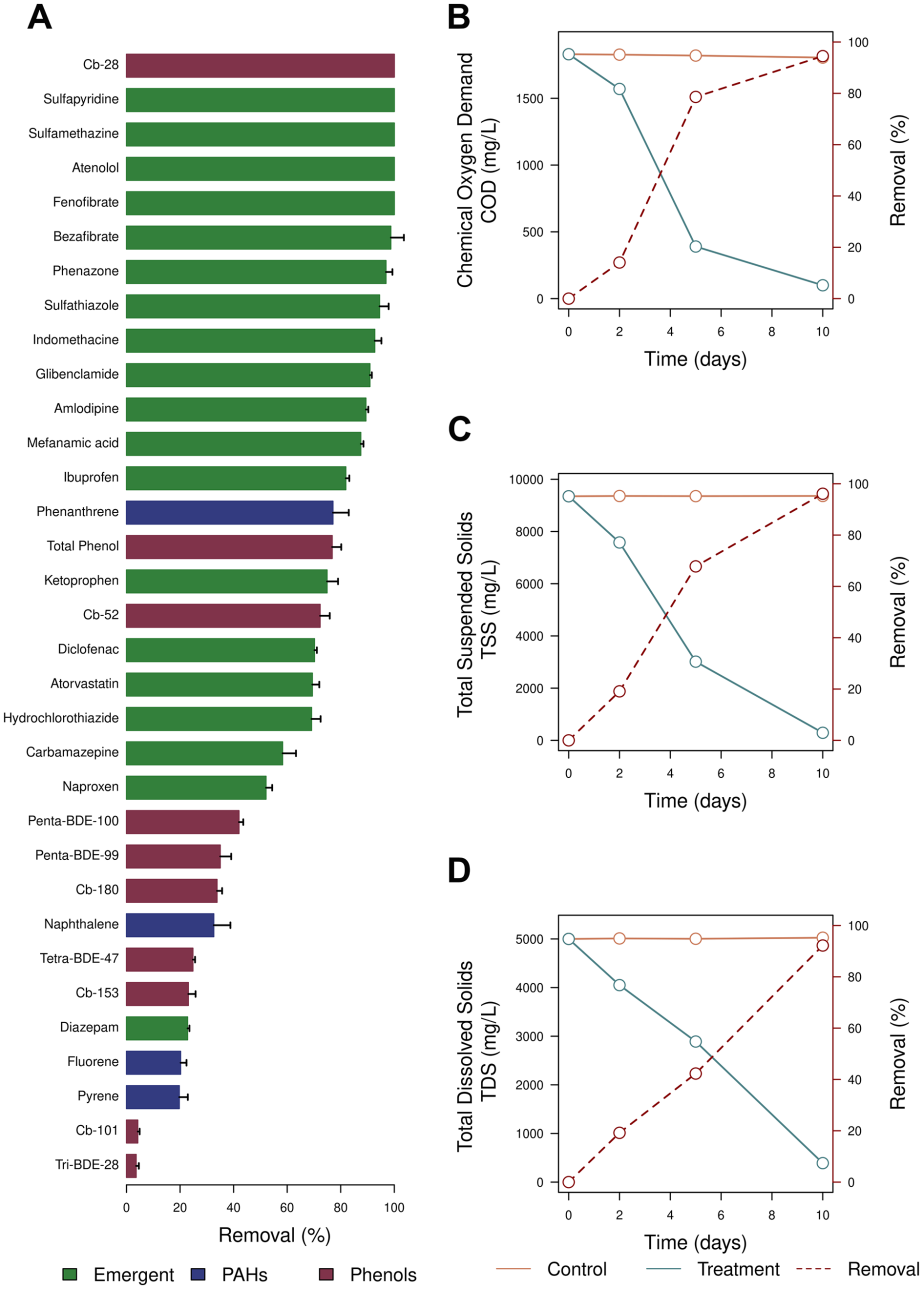
Inoculation of biosolids using *Aspergillus atacamensis* EXF-6660. **(A)** Removal of phenols, polycyclic aromatic hydrocarbons, and emergent pollutants. **(B)** Removal of the chemical oxygen demand (COD). **(C)** Removal of the total suspended solids (TSS). **(D)** Removal of the total dissolved solid (TDS).

Finally, we evaluated the ability of *A. atacamensis* EXF-6660 for biosolid treatment. This halophilic fungus showed a great potential to remove xenobiotic compounds from biosolids (Figures 7A–D). Pharmaceutical active compounds including sulfonamide antibiotics, lipid regulators and cholesterol lowering statin drugs, β-blockers, analgesic, and anti-inflammatories were extensively removed (> 80% for all cases), when biosolids were inoculated with *A. atacamensis* (Figure 7A). Phenols (total) and other aromatic compounds such as phenanthrene were also removed around 75%, while naphthalene, pyrene, and fluorene decreased their concentrations between 20 and 30%. Interestingly, the removal percentages of the emergent pollutants 2,4,4′-trichorobiphenyl (Cb-28) and 2,2′,5,5′- tetrachlorobiphenyl (Cb-52) were 100 and 72.3%, respectively, while other such as 2,2′,4,4′,5,5′-hexachlorobiphenyl (Cb-153), 2,2′,3,4,4′,5,5′-heptachlorobiphenyl (Cb-180), 2,2′,4,4′-tetrabromodiphenyl ether (tetra-BDE-47), 2,2′,4,4′,5-pentabromodiphenyl ether (penta-BDE-99), and 2,2′,4,4′,6-pentabromodiphenyl ether (penta-BDE-100) were removed in the range 25–45% (Figure 7A). On the other hand, the addition of *A. atacamensis* to the biosolids drastically decreased the chemical oxygen demand (94.5%) and removed the 96.1 and 92.2% of the total suspended solids and total dissolved solids, respectively (Figures 7B–D).

## Discussion

In order to dig on the physiologic-, metabolic- and morphologic responses of *A. atacamensis* EXF-6660 when exposed to varying concentrations of different solutes and carbon sources, we performed the present study. Our results show that this obligate halophile is able to grow and produces phialides and conidia in the presence of varying concentrations of both kosmotropic (NaCl, KCl, and sorbitol) and chaotropic agents (MgCl_2_, LiCl, CaCl_2_, and glycerol) (Figures 1–3). We also found here that *A. atacamensis* EXF-6660 is metabolically versatile, being able to use a huge repertory of organic molecules when supplied as sole carbon sources, in the presence of high NaCl concentrations (Figures 4, 5). Our findings also show that strain EXF-6660 can degrade various xenobiotics (PAHs, benzene derivatives and dyes), as well as different carbohydrates, in liquid medium containing 2.0 M NaCl (Figure 6). Finally, we demonstrated that *A. atacamensis* EXF-6660 has a great potential for biosolid treatments because high removal percentages of phenols, PAHs and emergent pollutants were observed when the fungus was added to biosolids. Moreover, COD, TSS and TDS decreased markedly during biosolid mycotreatment (Figure 7). Altogether, these results shed light on the extreme versatility of *A. atacamensis* EXF-6660 metabolism and emphasize its potential as a promising candidate for bioremediation or biotechnological purposes.

Although the obligate halophily is extremely rare in fungi, some aspergilli such as *A. baarnensis, A. salinarus* (Greiner et al., 2014), *A. destruens* (Sklenář et al., 2017), *A. salisburgensis*, and *A. atacamensis* (Martinelli et al., 2017) require NaCl to grow. Certain fungi are also known for their ability to grow not only at extremely high concentrations of the kosmotropic (stabilizing) salt NaCl but also in the presence of others kosmotropes (KCl and MgSO_4_) as well as in the presence of CaCl_2_, MgCl_2_, NaBr, and LiCl that act as chaotropic (destabilizing) salts (Zajc et al., 2014a). Although fungi can grow in habitats such as crystallizer ponds of solar salterns (Butinar et al., 2005a,b; Cantrell et al., 2006; Gunde-Cimerman et al., 2006), the Dead Sea (Oren and Gunde-Cimerman, 2012), and the brine channels in Artic subglacial ice (Gunde-Cimerman et al., 2003; Sonjak et al., 2006, 2010) where the ratio between chaotropic and kosmotropic ions is high, the characterization of kosmotolerant and chaotolerant fungi is scarce and should be expanded. Understanding the boundaries of life of obligate halophilic fungi at high concentrations of chaotropic salts, particularly in the presence of those containing divalent ions, is also of great value for evolution and paleobiology studies, but also for astronomical-biological sciences because Mg^2+^- and Ca^2+^-rich environments are prevalent on Mars (Cockell, 2020).

*Aspergillus atacamensis* EXF-6660, an obligate halophilic phialosimplex-like ascomycete isolated from a salt water-exposed cave in the hyperarid Atacama Desert in Chile (Martinelli et al., 2017), showed an elevated tolerance to extremely high concentrations of the kosmotropes NaCl and KCl, and the chaotropes CaCl_2_, MgCl_2_ and LiCl (Figures 1–3). The cell clumps forming fungus *W. ichthyophaga*, the black yeast *H. werneckii* and the filamentous fungi *Eurotium repens* and *Cladosporium cladosporioides* have also shown the ability to grow on salts that act as kosmotropes (NaCl, KCl, and MgSO_4_) and chaotropes (CaCl_2_, MgCl_2_, and NaBr) (Zajc et al., 2014a). *A. atacamensis* also exhibited high tolerance to sorbitol and glycerol, which considerably limit the water availability when present at high concentrations in media. *A. atacamensis* as well as *Xeromyces bisporus* (Pitt and Hocking, 2009; Williams and Hallsworth, 2009) had preference to grow in highly xerophilic media containing 2.5 and 5.13 M of sorbitol and glycerol, respectively (Figures 1–3). *X. bisporus* was described as the first fungus that showed extremely high tolerance to chaotropic conditions, being able to grow in the presence of 7.6 M of the chaotropic osmolyte glycerol (Williams and Hallsworth, 2009). However, unlike *A. atacamensis*, *X. bisporus* is intolerant to NaCl (Pitt and Hocking, 1977). *A. atacamensis* did not only tolerate but also thrive at high concentrations of all tested salts and solutes, even in the presence of those chaotropic salts which are more toxic to biological systems (Hofmeister, 1888; Kunz et al., 2004; Cray et al., 2015; Figures 1–3). Thus, the obligate halophilic *A. atacamensis* EXF-6660 can be considered an extremely chaotolerant, kosmotolerant, and xerotolerant fungus. This research adds to our understanding of how life performs in the presence of various kosmotropes and chaotropes, some of which, like chaotropic salts, produce a significant water-mediated cellular stress by entropically disarraying biomolecules (Hallsworth, 2022).

Our results also revealed that *A. atacamensis* EXF-6660 produced spores in the presence of varying concentrations of both kosmotropic (NaCl, KCl, and sorbitol) and chaotropic agents (MgCl_2_, LiCl, CaCl_2_, and glycerol). These results agree with those published by Zajc et al. (2014a) who studied many species of *Aureobasidium* (*A. pullulans, A. melanogenum, A. subglaciale*, and *A. namibiae*), *Hortaea* (*H. werneckii*), *Wallemia* (*W. ichthyophaga, W. muriae*, and *W. sebi*), and the cosmopolitan genera *Cladosporium* (*C. cladosporioides* and *C. tenuissimum*) and *Penicillium* (*P. chrysogenum, P. commune*, and *P. stecki*) that exhibited both variable tolerance to different salts and the formation of reproductive structures under chaotropic and kosmotropic conditions.

Interestingly, *A. atacamensis* EXF-6660 was able to grow up to saturating levels of NaCl, with a broad optimum from 1.5 to 3.4 M NaCl (Figure 1). As expected, while no mycelial growth was observed in the absence of salt, this fungus grew better in NaCl-rich media (Martinelli et al., 2017). These results reflect that the phialosimplex-like *A. atacamensis* as well as the basidiomycetous *W. ichthyophaga* could be consider the most halophilic fungi so far described (Gostinčar et al., 2011, 2022; Zajc et al., 2013, 2014b; Gunde-Cimerman et al., 2018). *A. atacamensis* grew in the whole range of NaCl concentrations like is observed in the melanized yeast *H. werneckii* (Gostinčar et al., 2011). Six species of the known teleomorphic food-borne xerophilic genus *Eurotium* (*E. amstelodami, E. chevalieri, E. halotolerans, E. herbariorum, E. repens, E. rubrum*) isolated from the Slovenian salterns Sečovlje located at the northern Adriatic coast, also showed a broad salinity growth range, from 0 M up to 4.7 M NaCl (Butinar et al., 2005c).

Surprisingly *A. atacamensis* EXF-6660 tolerated high concentrations of MgCl_2_ (Figures 1, 2). This fungus was able to grow up to 2.0 M of this chaotropic salt and even survived at 2.5 M MgCl_2_, although not succeeded by growth at this salt concentration. It is known that microbial life is strongly inhibited at ≥1.26 M of MgCl_2_ and seems to be completely abolished at 2.3 M MgCl_2_ because mRNAs are thermodynamically instable at higher concentrations of Mg^2+^ (Hallsworth et al., 2007; McGenity and Oren, 2012). Thus, *A. atacamensis* is an obligate halophilic fungus that grows in the presence of much higher concentrations of MgCl_2_ than previously reported to be the upper limit (1.26 M) to support prokaryotic life. The halotolerant fungi *H. werneckii, W. muriae*, *W. sebi*, and some species of *Aspergillus, Cladosporium, Emericella, Eurotium, Penicillium, Phaeotheca, Trichosporon,* and *Ulocladium* were also able to grow in media containing ≥1.8 M MgCl_2_ without compensating kosmotropic salts (Sonjak et al., 2010; Zajc et al., 2014a). Many strains of the halophilic fungus *W. ichthyophaga* have showed unique phenotypes to tolerate ≥2.0 M MgCl_2_, but not CaCl_2_ (Zajc et al., 2014a). *A. atacamensis* was also able to grow under chaotropic conditions induced by high concentrations of CaCl_2_ (> 1.0 M). This is comparable to what occurs with other aspergilli, such as *A. caespitosus, A. flavipes, A. proliferans, A. sclerotiorum, A. sydowii, A. tubingensis,* and *A. ustus*, and the melanized yeast *H. werneckii* (Zajc et al., 2014a). These authors characterized the growth of 135 fungal strains from 94 species and 31 genera and discovered that only two species, *A. flavipes* and *A. ustus*, grew at 2.0 M CaCl_2_ (Zajc et al., 2014a). However, the growth of *A. atacamensis* was observed up to 2.5 M of CaCl_2_. The physiology and molecular biology of the microbial growth in Mg^2+^- and Ca^2+^-rich media remain poorly understood yet.

Although the growth of *A. atacamensis* was drastically inhibited by LiCl, tiny colonies were observed in the range from 0.5 to 1.5 M LiCl (Figure 1). It is known that Li^+^ significantly affects the viability of certain fungi such as *A. niger, P. chrysogenum*, and *Penicillium simplicissimum* at 5.9 mM (250 mg/L) (Lobos et al., 2021). The growth of other species such as *Chaetomium globosum* and *Postia placenta* was abolished at 70 mM (3,000 mg/L) LiCl, while *A. niger* tolerated 140 mM (6,000 mg/L) LiCl (Richter et al., 2008). Thus, *A. atacamensis* has a higher tolerance to Li-ions in comparison to other fungal species. This is particularly interesting for Li-bioleaching from spent rechargeable Li-ion batteries and to recover Li as a valuable metal from electronic wastes (Moazzam et al., 2021).

In *A. atacamensis* the isotropic growth and swelling of hyphae was more noticeable in the presence of MgCl_2_ (0.5 M), sorbitol (1.0 M), and glycerol (4.2 M) in comparison with the other conditions studied. Swelling is a process that occurs during the first stage of conidial germination in the genus *Aspergillus* (Van Leeuwen et al., 2010). In response to stress conditions, extremophilic fungi produced swollen hyphae. This was demonstrated for *Acidomyces acidophilus*, an acidophilic fungus formerly known as *Scytalidium acidophilum* (Selbmann et al., 2008), and the halophilic *A. sydowii* (Peidro-Guzmán et al., 2021), when grown in the presence of 0.5 M HCl and PAHs, respectively. In the presence of 1.7 and 5.13 M NaCl, *A. atacamensis* showed smallest phialides and, conidia and chlamydospore with larger sizes of length and width compared to those found in *Aspergillus chlamydosporum, A. salinarus* and *A. salisburgensis* in the range from 5% (0.85 M) to 10% (1.7 M) NaCl (Sigler et al., 2010; Greiner et al., 2014; Martinelli et al., 2017).

The exceptional ability of *A. atacamensis* to grow at high concentrations of different chaotropes and kosmotropes could be related to the presence of genes encoding for a huge repertory of plasma membrane ion transporters. Also, this phenotypic plasticity to survive on salts-dominated media might involve a rigorous transcriptional reprogramming that leads to subsequent synthesis of compatible solutes, a fine tuning of intracellular ion concentrations, changes in the composition and functionality of the cell membranes and importantly, a severe remodeling of the fungal cell wall to modify the relative abundance of its different components and the cell-wall ultrastructure and morphology.

Although the life-limiting effect of NaCl at high concentrations, *A. atacamensis* EXF-6660 showed a great biochemical plasticity evidenced by a versatile metabolism at 0.85 M (5%) and 2.0 M (12%) NaCl (Figures 4, 5). As we have shown, while 0.85 and 2.0 M of NaCl did not affect the metabolic activity of *A. atacamensis* because it was able to utilize a range (≥ 95%) of carbon substrates in the Biolog plates, saturated NaCl concentrations (5.13 M, 30%) had an inhibitory effect (Figure 4). At this salt concentration only ~50% of the carbon sources were metabolized (Figure 4). At optimal (12%) and sub-optimal (0.85%) salinities, many substrates of the tricarboxylic acid cycle and the pentose phosphate pathway were highly utilized, suggesting that these biochemical pathways probably contribute indistinctly to the energy production in *A. atacamensis* when grown at these NaCl concentrations. In contrast, the preferential consumption of the substrates involved in the pentose phosphate pathway at saturated NaCl concentrations could suggest that this biochemical pathway is the main way for energy production in *A. atacamensis* when exposed to extremely high salinity (Wang et al., 2016b). The number of carbon sources metabolized by *A. atacamensis* at optimal and sub-optimal conditions (≥ 95%) was similar than that of the plant pathogen *Botrytis cinerea* (96.8%) (Wang et al., 2016b). Also, four different strains of the cosmopolitan fungus *A. niger* also revealed high significant metabolic diversity (Šimonovičová et al., 2020). In comparison, the polyextremophile fungus *Aspergillus terreus* NTOU4989, isolated from sulfur sediment collected at hydrothermal vent field, was able to utilize seventy-one (71/95) of the carbon substrates present in the Biolog plates, at a NaCl concentration of 3% (~0.5 M) (Chou et al., 2022). An isolate closely related *to A. sydowii* utilized only thirty-seven (37/95) carbon sources in the absence of salt (Yu et al., 2007), while the plant pathogen *Fusarium kyushuense* metabolized eighty-seven (87/95) of the substrates included in the Biolog test system (Wang et al., 2016a). These results remark the exceptional metabolic versatility of *A. atacamensis* at low water activity conditions, even in comparison to other aspergilli.

Finally, this work highlights the potential of *A. atacamensis* EXF-6660 for the implementation of different biotechnologies. The results presented here demonstrate that *A. atacamensis* also possesses a substantial repertoire of enzymes allowing it to metabolize a diversity of complex and model substrates (Figures 4–6) at high salinity conditions.

At optimal salinity conditions, *A. atacamensis* EXF-6660 was able to degrade complex substrates like cornstarch, cellulose, lignin, and their monomers xylose, maltose and even some phenolic byproducts including gallic acid, phenol and guaiacol (Figure 6). All these compounds are related to lignocellulosic biomass, one of the most relevant materials used in the second and third generation of biofuel production technologies. Therefore, the enzymes produced by *A. atacamensis* are good candidates to pretreat lignocellulosic wastes or even microalgal biomass to produce biofuels (Zabed et al., 2019). Crude enzymatic cocktails of *Aspergillus lentus* have been previously used to improve the solubilization of sugars present in microalgal biomass with promising results (Prajapati et al., 2015). During the degradation of lignocellulosic materials, a variety of aromatic monomers are released as intermediates: for example, ferulic acid, phenol, guaiacol, vanillin and gallic acid among many others (Lubbers et al., 2019). To date, most of the bacterial enzymes and metabolic pathways related to aromatic compounds have been characterized; surprisingly, only a few of these enzymes have been characterized in fungi and yeasts (Mäkelä et al., 2015; Lubbers et al., 2019). Our results revealed that *A. atacamensis* EXF-6660 is able to utilize guaiacol, phenol and gallic acid making this obligate halophilic fungus a good candidate for the study of enzymes involved in the metabolism of aromatic compounds under conditions of low a_w_.

We also show that *A. atacamensis* EXF-6660 was able to grow in the presence of xenobiotics including dyes, PAHs, pesticides (i.e., piperonyl butoxide) and phthalates (Figure 6). All these compounds represent frequent pollutants of aquatic ecosystems, since they are very often released through effluents by different factories and manufacturers, either accidentally or as by-products of their processes (Khan et al., 2019; Ali et al., 2021). Likewise, there are previous reports of phthalates (Carstens et al., 2020) and PAHs (Alao and Adebayo, 2022) metabolization by fungi. However, the use of halophilic fungi to degrade pollutants is still in its very first stages (González-Abradelo et al., 2019; Martínez-Ávila et al., 2021; Peidro-Guzmán et al., 2021) and has a very promising future (Śliżewska et al., 2022).

On the other hand, the textile industry is avidly seeking for enzymes such as laccases, lignin peroxidases and manganese peroxidases, which are useful to degrade and decolorize the dyes it uses and releases to the effluents (Pérez-Llano et al., 2020b; Kalra and Gupta, 2022). Until now, many genera of fungi have been employed to decolorize a wide range of dyes including species of the *Aspergillus* genera (Pan et al., 2017; Dhir, 2022). *A. atacamensis* EXF-6660 could be added to this list of microbial sources of dye-degrading enzymes, active at high concentrations of salts and other organic compounds.

In addition, *A. atacamensis* EXF-6660 showed a significant metabolic activity on biosolids (Figure 7). The removal pattern of the individual PAHs, pharmaceutical active compounds, phenols, biphenyls, and diphenyl ether compounds reveals the great potential of this fungus for biosolid mycotreatment. The levels of pollutant removal observed for *A. atacamensis* were similar that those found for *Phanerochaete chrysosporium* (Taha et al., 2018), *Trametes hirsuta* (Saibi et al., 2022), and *Trametes versicolor* (Rodríguez-Rodríguez et al., 2011, 2012, 2014). The reduction of the COD (94.5%), TSS (96.1%), and TDS (92.2%) emphasizes the utility of *A. atacamensis* EXF-6660 to develop eco-friendly technologies for biosolid treatments. Thus, we propose that, among the multiple potential uses of *A. atacamensis* EXF-6660, it can be considered as a very promising agent to develop innovative bioremediation strategies, aimed at removing toxic xenobiotics.

## Conclusion

This study analyzed how the chaotropic and kosmotropic conditions of the media affect the growth and metabolism of the obligate halophilic fungus *A. atacamensis* EXF-6660. Remarkable changes in the morphology of *A. atacamensis* were observed when the fungus was grown at different concentrations of both chaotropic (MgCl_2_, LiCl, CaCl_2_, and glycerol) and kosmotropic (NaCl, KCl, and sorbitol) agents. The phialosimplex-like *A. atacamensis* EXF-6660 was metabolically versatile, being able to use a huge repertory of organic molecules when supplied as sole carbon sources, in the presence of high NaCl concentrations. Our findings also showed that strain EXF-6660 can degrade various xenobiotics (PAHs, benzene derivatives and dyes), as well as different carbohydrates, in culture media containing 2.0 M NaCl. Finally, we demonstrated that *A. atacamensis* EXF-6660 has a great potential for biosolid treatments because high removal percentages of phenols, PAHs and emergent pollutants were observed when the fungus was added to biosolids. Altogether, these results shed light on the extreme versatility of *A. atacamensis* EXF-6660 metabolism and emphasize its potential as a promising candidate for bioremediation or biotechnological purposes. Understanding the life-limits of obligate halophilic fungi at high concentrations of chaotropic and kosmotropic salts is important for studying not only evolution and paleobiology, but also astronomical-biological sciences, the development of novel preservation techniques for the fungal diversity of salt-dominated extreme environments, and the implementation of leading-edge biotechnologies.

## Data availability statement

The original contributions presented in the study are included in the article, further inquiries can be directed to the corresponding author.

## Author contributions

LY, TM-P, GV-M, and RB-G wrote the first draft of the manuscript. TM-P, GV-M, and RB-G prepared the figures. All authors commented on previous versions of the manuscript, contributed to the study conception, design, investigation, read, and approved the final manuscript.

## Funding

RB-G received a Sabbatical fellowship (CVU: 389616) from the National Council of Science and Technology (CONACyT), Government of Mexico. This work was supported by Fondo Nacional de Innovación y Desarrollo Científico-Tecnológico (FONDOCYT), Ministerio de Educación Superior, Ciencia y Tecnología (MESCYT), Government of Dominican Republic: Project COD. 2022-2B2-078. This work was partially supported by CONACyT, Government of Mexico: Projects CONACyT 1059 and 315114, Project CONACyT-SEP CB-285816. This work was also supported by funding from the Slovenian Research Agency to Infrastructural Centre Mycosmo (MRIC UL, I0-0022), programs P4-0432 and P1-0198.

## Conflict of interest

The authors declare that the research was conducted in the absence of any commercial or financial relationships that could be construed as a potential conflict of interest.

## Publisher’s note

All claims expressed in this article are solely those of the authors and do not necessarily represent those of their affiliated organizations, or those of the publisher, the editors and the reviewers. Any product that may be evaluated in this article, or claim that may be made by its manufacturer, is not guaranteed or endorsed by the publisher.
